# The needs, models of care, interventions and outcomes of palliative care in the Caribbean: a systematic review of the evidence

**DOI:** 10.1186/s12904-016-0079-6

**Published:** 2016-01-22

**Authors:** Sandhya Maharaj, Richard Harding

**Affiliations:** Caura Palliative Care Unit, Caura Hospital, El Dorado, Trinidad and Tobago; Department of Palliative Care,Policy and Rehabilitation, Cicely Saunders Institute, King’s College London, Bessemer Road, London, SE59PJ UK

**Keywords:** Palliative care needs, Models of care, Interventions, Outcomes, Caribbean, Systematic review

## Abstract

**Background:**

Palliative care provision is expanding in low and middle income countries. Services are developing in the Caribbean in response to the region’s ageing population, the significant burden of cancer, non-communicable diseases and HIV/AIDS. Appraisal of the existing evidence on palliative care needs, models of care, interventions and outcomes in the Caribbean is essential to inform emerging practice and future research.

**Methods:**

Systematic review and narrative synthesis. Following implementation of a search strategy, titles, abstracts and full texts were screened. Data from nine studies were synthesized. The Qualsyst tools were used to assess the quality of quantitative and qualitative studies. Data were extracted into a common table, and themes were generated from the available peer review evidence using narrative synthesis.

**Results:**

Nine papers were retained for appraisal. Eight papers described palliative care needs in the Caribbean. The needs for analgesia, support for patients, education and training of staff in palliative care and palliative care services were identified in the literature. Models of care for palliative care in the Caribbean were not described in great depth (*n* = 2 papers) and no intervention studies were found. Outcomes of palliative care such as quality of life, quality of care, and patient’s preferred place of care and death were identified from six papers. Quantitative methodology was used in seven of the nine papers in this review. One paper used a mixed methodology design, and one a qualitative approach.

**Conclusions:**

Research from the Caribbean highlights the need for health care policy, training of staff, education, and access to analgesia and palliative care support services in this region. This sparse evidence must be taken into consideration with cultural beliefs and preferences of the Caribbean population in order to achieve improved outcomes for patients, their caregivers and health care professionals. This underscores the importance for more research in the field of palliative care in the Caribbean.

## Background

Of the 58 million people who die yearly, 45 million die in low and middle income countries (LMIC) [1]. In the last decade, 99 % of the population growth took place in the developing world [2]. It is expected that by 2050, the population in developing countries will expand by 55 % [2]. The Caribbean is defined as the Caribbean islands that are located in the Caribbean sea. These islands are Anguilla, Antigua and Barbuda, Aruba, Barbados, Bonaire, British Virgin Islands, Curaçao, Dominica, Grenada, Guadeloupe, Martinique, Montserrat, Saba, Saint Barthelemy, Saint Kitts and Nevis, Saint Lucia, Saint Martin, Saint Vincent and the Grenadines, Saint Eustatius, Trinidad and Tobago, US Virgin Islands, Cuba, Hispaniola (Haiti and the Dominican Republic), Jamaica, Puerto Rico, the Cayman Islands, the Bahamas and the Turks and Caicos Islands. These islands form an estimated population of 32 million persons [3, 4]. The Pan American Health Organization (PAHO) database states the leading causes of death in the Caribbean islands from 1995 to 2010 as Ischaemic Heart Disease, Cerebrovascular Disease, Diabetes Mellitus, Influenza and Pneumonia, Malignant neoplasm of the trachea, bronchus and the lung, Hypertensive diseases, Chronic diseases of the lower respiratory tract, Heart failure and ill-defined heart disease, Malignant neoplasm of the prostate and Dementia [5]. The majority of these top ten causes of mortality require palliative care at some point during their illness trajectory [6].

In 2013, PAHO/WHO (World Health Organization) reported that 50 % of the cancer deaths in the Americas come from Latin America and the Caribbean [7]. The highest cancer mortality rates in the region are found in Trinidad and Tobago and Cuba [7]. Between 2012 and 2030 there will be an expected 67 % rise in cancer incidence in the Caribbean and Latin America [8]. It is projected that by 2030, there would be 1.8 million people diagnosed yearly with cancer in the Caribbean and Latin America. In addition, nearly 80 % of deaths from non-communicable diseases (NCDs) occur in low income and middle income countries [9]. In Latin America and the Caribbean specifically, chronic diseases are now the leading cause of premature mortality and account for two out of three deaths overall [10]. The Caribbean is the region of the Americas affected the most by the epidemic of chronic diseases [10].

The Joint United Nations Programme on HIV and AIDS (UNAIDS), suggest that after Sub-Saharan Africa, the Caribbean is one of the most heavily affected regions with the Human Immunodeficiency Virus (HIV) epidemic [11]. The adult HIV prevalence in 2011 was documented as 1 %, higher than any other region in the world apart from Sub- Saharan Africa [11].

The WHO projects greater life expectancy for the population of Latin America and Caribbean countries between 2004 and 2030 [12]. This gain in life expectancy brings with it complex demands on the health care system [12]. The health care needs of the elderly may be chronic, time consuming and costly [12, 13].

With the morbidity and mortality trends seen in Latin America and the Caribbean, it is estimated that approximately a half million people require palliative care services [14]. The latest global categorisation of countries in the Caribbean in terms of their palliative care integration into health systems is as follows: Group 1 (no known activity) *n* = 10; Group 2 (capacity building activity only) *n* = 4; Group 3a (isolated provision) *n* = 8; Group 3b (generalised provision) *n* = 0; Group 4a (preliminary integration into mainstream) *n* = 1; Group 4b (integrated provision) *n* = 0 [15].

There appears to be a discrepancy between the need for, and existence of, palliative care services in the Caribbean, since the majority of Caribbean islands have no or only isolated palliative care provision. A bibliometric analysis looking at research activity in palliative care in Latin America and the Caribbean (LAC) found that there was a modest contribution of research from LAC to international palliative care literature [16].

LMIC have conducted and reported systematic reviews to appraise the current state of evidence, and synergise research activities to improve the availability and quality of palliative care [17–19]. This study aimed to identify and appraise the evidence needs, models of care, interventions and outcomes of palliative care in the Caribbean.

### Research question

A systematic review of the literature was conducted to answer the question:

“What are the needs, models of care, interventions and outcomes of palliative care in the Caribbean?”

## Methods

### Design statement

A systematic literature review was conducted in line with the Preferred Reporting Items for Systematic Reviews and Meta-Analyses (PRISMA) [20] statement and the guidelines set out from the National Health Service (NHS) Centre for Reviews and Dissemination (CRD) [21]. The synthesis of the retained data was carried out using the narrative synthesis framework by Popay et al [22]. There were no ethics required as this was a systematic review of published literature.

### Definitions

The definitions of terms used in this review are presented in Table 1.

**Table 1:** Definitions of terms used in the systematic review

Palliative care Patient	The term “palliative” when used to describe patients, covers a varied heterogeneous population [45]. Patients with certain primary diseases have a palliative period, the period when the disease has become progressive and emphasis is not placed on cure but quality of life that follows [45].
Health Care needs	Health care need can be defined as the “capacity to benefit from health care” [46]. The WHO further defines it as perceived health care needs as experienced by the patient, professionally defined health care needs as those services defined by health professionals and scientifically confirmed needs [47].
Models of Care	A model for Palliative care [48]:
	• Provides services for patients with life limiting illness regardless of diagnosis
	• Addresses the palliative care needs of patients and their families during their illness trajectory
	• Delivers care in any setting-hospital, palliative care unit, residential care or home
	• Identifies partnerships between specialist palliative care services and primary care providers
Palliative care Interventions	“Palliative interventions aim to relieve suffering and improve quality of life for those who are living with, or dying from, a terminal/advanced illness.” [49]
Palliative care outcomes	A health outcome is defined as “*Change in a patient’s current or future health status that can be attributed to antecedent health care”*[50]
End of Life	The General Medical Council (GMC) defines patients as “approaching the end of life” when they are likely to die within the next twelve months [51]. This is broadly accepted cross-nationally [52].
Advanced disease	“Connected with the active and progressive disease and has a limited prognosis. Prognostication in advanced disease relates to different factors such as symptoms, performance status and disease trajectory. As disease trajectories vary depending on whether the patient is suffering from malignant or non-malignant disease [53], advanced stages have to be defined independently for every disease”[54]

### Search strategy

The biomedical databases of MEDLINE, EMBASE, CINAHL, Web of Science and Global Health were searched from 1987 (the recognition of palliative care as a specialty in medicine) to October 2014.

Search terms used were the union of the two groups of search terms as represented in Table 2. The first search strategy was the separate run of group 1 and group 2’s search terms. The second search strategy was the union of group 1 and group 2 results with ‘and’.

**Table 2:** The search strategy used in the databases

Search terms Group1	Search terms Group 2
• Palliative care OR	• Caribbean OR
•Terminal* OR	• West Indies OR
• Terminally Ill OR	• Commonwealth Countries OR
• Terminal Care OR	• Each named Caribbean Island: Anguilla, Aruba, Antigua and Barbuda, Barbados, Bonaire, British Virgin Islands,Curaçao,Dominica,Grenada,Guadeloupe,Martinique,Monserrat,Saba,Saint Barthelmey, Saint Kitts and Nevis, Saint Lucia, Saint Martin, Saint Vincent and the Grenadines, Saint Eustatius, Trinidad and Tobago, US Virgin Islands, Cuba, Haiti, Dominican Republic, Jamaica,Puerto Rico, Cayman Islands, The Bahamas and Turks and Caicos
• Hospice OR	
• End Of Life OR	
• Dying OR	
• Advanced Disease OR: HIV/AIDS OR Chronic Heart Failure OR Advanced COPD (Chronic Obstructive Lung Disease) OR Chronic Renal Disease OR Dementia OR Advanced Cancer (these groups were used since they fall into the top causes of mortality in the Caribbean)	

Apart from database searching, key experts in the field of palliative care in the Caribbean were contacted to enquire about relevant published research. These persons were sourced by the regional palliative care societies, a search of contacts from the website page of the International Association for Hospice and Palliative care, and recommendations of health professionals from the Caribbean.

The Caribbean Medical Journal, The Journal of Palliative Care and the West Indian Medical Journal were all hand searched because no online versions were available. A supplemental online search was also performed of the West Indian Medical Journal. This engagement with the literature started in July 2014 by hand searching the Journal of Palliative Care. Searching of the electronic databases and hand searching of the other two journals was conducted in October 2014.

Once the literature search was completed, all the articles were exported to the bibliographic software tool Endnote Version X7.1 for the removal of duplicate articles and then scrutiny of the remaining articles.

### Inclusion and exclusion criteria

Both qualitative and quantitative research papers were included for analysis. The criteria used for selection of the studies are demonstrated in Table 3.

**Table 3:** Inclusion and exclusion criteria for studies in the systematic review

Inclusion criteria	Exclusion criteria
Peer review publications including data from at least one of the Caribbean islands addressing palliative care needs, models of care, interventions and outcomes in the Caribbean	Case Studies, Reviews or non-peer review documents
Papers in English	Studies reporting data from people of Caribbean origin outside of the Caribbean setting
Papers reporting adult subjects only: patients, caregivers or health care practitioners	Conference Abstracts
Papers dealing with progressive incurable conditions	Papers on frailty and ageing
Data reporting early stages of a progressive condition except HIV/AIDS and Dementia.	

A first researcher (SM) screened all full abstracts to assess for eligibility against the inclusion/exclusion criteria. The full text of eligible articles was retrieved if the paper met inclusion criteria, or if the abstract did not contribute adequate detail to warrant rejection. If there was any uncertainty after the full text of the article was examined, a second researcher (RH) assisted in making the final decision on agreement for inclusion of the paper.

### Data extraction

Data extraction is required to accurately extract the relevant features and the results of the selected studies [23]. Data were extracted by one researcher (SM) and reviewed by a second researcher (RH) if necessary.

Publication details, study aim, sample characteristics, and main findings were extracted from each retained paper into a common table.

### Quality assessment

Quality assessment allows both the internal validity and external validity of a study to be considered [23]. The quality assessment tools called the QualSyst tools [24], constructed by researchers from the Alberta Heritage Foundation were used for assessment of the quality of both qualitative and quantitative papers using any study design.

A numerical final score derived from a scoring system provides a systematic, reproducible and quantitative means of assessing the quality of research amongst different study designs [25].

The Qualsyst tool for assessment of the quality of quantitative studies is a checklist of 14 questions. A score from 0 to 2 can be awarded to each item on the tool. A summary score is calculated for each paper when the tool is applied. The Qualsyst tool for assessment of the quality of quantitative studies is a checklist of 14 questions. A summary score is calculated for each paper when the tool is applied by calculating a total sum then dividing it by the total possible sum [24]. The creators of the tool suggested that a cut off of 0.75 as a score to be the threshold for allowing a paper to be included in a review. The definition of the quality of a paper was defined as follows by Lee et al [25] in the use of this tool in their systematic review as; strong (summary score of >0.80),good (summary score of 0.71-0.79),adequate (summary score of 0.50-0.70) and limited (summary score of <0.50). The method of assessing the quality suggested by Lee et al [25] was appropriate for our goal of assessment of the quality of the data rather than using a threshold for including a paper into the review.

The Qualsyst tool for assessment of qualitative studies is a validated checklist that is made up of 10 criteria to be assessed. The scores for each item range from 0 to 2 with a maximum total score being 20. A summary score can be attained by summating the total score across the ten items and dividing it by 20. A threshold of 0.55 was used by the creators for inclusion of qualitative studies into their systematic review [24]. For the purposes of this review, the researcher defined a score of ≥0.55 as an ‘adequate quality’ paper. A score of ≤0.54 was deemed as a ‘low quality’ paper.

### Data synthesis

The method of data synthesis used for this systematic review was narrative synthesis. This method was chosen because of the decision to include both qualitative and quantitative data in the final analysis. The guidance from Popay et al [22] allows synthesis of a wide array of research designs. The general framework for conducting narrative synthesis as suggested by the authors, Popay et al, consists of four main elements [22]:

1. Element 1: developing a theory of how the intervention works, why and for whom. A theory was not developed for this systematic review as all of the studies were non-intervention studies and descriptive in nature that were synthesized.
2. Element 2: developing a preliminary synthesis of findings of included studies. There are different tools and techniques for preliminary synthesis of findings. One tool of tabulation was used in the form of data extraction table as presented in Table 4. In addition to tabulation, thematic analysis was also performed. The school of Psychology at the University of Auckland proposes six steps for conduct of thematic analysis [26] which were applied in this review. These steps include familiarization with the data, coding, searching for themes, reviewing the themes, defining and naming the themes and then producing the report.
3. Element 3: exploring relationships in the data. Idea webbing and concept mapping were used to analyse the findings from this review.
4. Element 4: assessing the robustness of the synthesis. Critical reflection of this review process was highlighted in the discussion section of this paper.

This framework allowed the researcher to carefully synthesize the data.

**Table 4:** Data Extraction Table

Study/Caribbean Island	Aim and design	Participants/setting	Main findings	Quality score
Hariharan et al [27] 2003 Barbados	To analyze the characteristics of moribund patients in a surgical intensive care unit (ICU) and highlight the dilemmas inherent in treating such patients. Cross Sectional Survey	*n* = 30, patients in a surgical ICU, deemed to be undergoing futile treatment	The majority of patients, even after the futility of treatment was established, withdrawal of any form of therapy was not attempted. Factors influencing the decisions on futility of care were: age of the patient, legal considerations, family influences and surgeon’s refusal to accept futility in a post operative patient. **Age:** Withdrawals of no forms of therapy were made in seven brain dead patients under the age of 30 years. In eight patients who were unresponsive after laparotomy, the four patients above 80 years, some form of therapeutic intervention was withheld. In the four patients who were in their sixties no support was withdrawn. **Legal considerations** Medico legal cases received more therapeutic support than non medicolegal cases. **Family influences** In 24 cases where family had hopes of their relatives recovery and requests to surgeons contributed to the maintenance of therapy. In two cases, there was fear of litigation on withdrawal of therapy since the patient’s relatives live abroad. Surgeons refusal to accept futility of care in the post operative patient. Support was continued on 12 patients based on the surgeons inability to accept futility of care	Quantitative score:0.80
Kreitzschitz et al [28] 2003 Grenada	To explore the end of life concerns in the Caribbean among health professionals and people who had lost a loved one. Qualitative Study	*n* = 32 (24 caregivers and 8 health professionals)	**Caregivers** More than half of the deaths of the patients occurred at home as reported by the caregivers. The themes uncovered were: *Place of death* The participants revealed that many of the deceased preferred to remain at home. *Pain and Suffering* Participants responses to pain and suffering of their deceased relative included: “pain and suffering is a must”, “there was a time when I thought she (my wife) was going to die because she had so much pain”, “bore her pain well, but whenever she was in great pain you would hear it”, “he was afraid of the pain” and some spoke about emotional pain the deceased expressed due to regrets, becoming a burden or loss of independence. *Sources of support for the deceased and their caregivers* Participants were unaware of the ways by which dying patients or their families could obtain counselling, financial assistance or help with home care. Almost all participants mentioned spirituality as a means of support *Use of herbal or traditional medications* No participants reported the use of traditional medicine near the end of life. *Concerns about end of life care* Many caregivers mentioned financial debts from medicines, treatments and/or the funeral. Participants complained about nurses, stating: “some of them were very harsh…my sister is a nurse. Every time she was there they treat him better…well everybody work under stress, not enough pay. I don’t blame them”. **Health professionals narratives** Nurses thought most patients die at home. Expressed that assistance for dying patients is limited: “there is no real policy in place for that kind of thing”, “we do not have an official, like support services, but we do have a hospital chaplain” and “facilities need improvement, more social workers, improved treatment to extend lives, more readily available care. We give lots of pain medications. Sometimes we run out, but rarely.” Nurses thought they are not hostile but remarked: “patients don’t tend to follow rules. I make sure they follow the doctors prescription and maintain the patients’ visiting hours, and I don’t think they like that.. we are short staffed”, “with a dying patient we try and make the patient feel as comfortable as possible.” Others thought compassionate care is hindered by low salaries. **Physicians opinions** Thought home care is often better than hospital care due to lack of hospital resources. One doctor pointed out that although dying persons abroad receive more pain relief, Caribbean people tend to “believe that illness is a god given destination, so they don’t mind suffering. They believe that maybe it was something they did, and it is a question of almost purification before they go beyond. So they accept it.” They expressed concerns about nurses’ low salaries and long working hours. Nurse to patient ratio makes is difficult for nurses to see dying patients at home. Also, lack of cars for the nurses hence they may need to walk to a patient’s home which is time consuming.	Qualitative score:0.70
Torres Vigil et al [29] 2006 Cuba	To assess the quality of advanced cancer care in five Latin American countries. Cross Sectional Survey	*n* = 76 (health practitioners : physicians and nurses)	The policy barriers identified to optimal advanced cancer care were (ranked in order of top priority identified by respondents): 1. international restrictions on the import and export of medications 2. fear of diversion of opioids to illegal market 3. palliative care is not a priority in health care education 4. restrictive prescribing related laws and regulations 5. palliative care is not a priority in formulation of a health care policy. Quality of care, access to care and affordability of care were all assessed as good for the appropriateness of advanced cancer care.	Quantitative score:0.89
Torres Vigil et al [30] 2008 Cuba	To determine health care providers’ assessments of the quality of advanced cancer care in Latin American Medical Institutions: Cuba and to determine which factors were most predictive of the provider’s assessments of the quality of advanced cancer care in their institutions, Cross-sectional survey	*n* = 76 (health practitioners : physicians and nurses)	Availability of end of life services and access to cancer care in practice settings were identified as predictors of the quality of advanced cancer care in practice settings. Strong positive associations between the report of institutional availability of key opioid analgesics and higher quality of care ratings. The specialty of the provider was related with the quality of care ratings. Barriers to optimal cancer pain management predicted the quality of care ratings. Respondents who cited “medical staff reluctance to prescribe opiates” as a top barrier, provided quality of care assessments that were much lower than those who did not identify this as a top barrier. The use of the WHO three step analgesic ladder for cancer pain relief was positively associated with higher ratings of quality of care	Quantitative score:0.94
Justo Roll et al [31] 2009 Cuba	To explore the palliative care needs of Cuban patients with advanced cancer. Cross Sectional survey	*n* = 91 (patients)	The most burdensome complaints as assessed by the POS for patients were: • Wasted times on appointments • Patient anxiety • Family anxiety Metastases were significantly associated with a worse score for pain, personal anxiety and family anxiety. Patients who were aware of their diagnoses had better scores with regards to symptoms, patient anxiety, information and support. 66 % of patients with high pain scores were not receiving strong opioids. There was a discrepancy between the amount patients wished to know and the amount they were told.	Quantitative score:0.89
Gayle et al [32] 2009 Jamaica	To document Quality Of Life and the predictive factors in a cohort of patients with End Stage Renal Disease in Jamaica. Cross Sectional Survey	*n* = 110 (*n* = 70 patients from a tertiary hospital based outpatient haemodialysis centre (UHWI) in Kingston, Jamaica, *n* = 40,patients with end stage renal failure from a privately owned haemodialysis centre (DARU) in Kingston, Jamaica)	Self administered Kidney Disease Quality of Life –Short Form Questionnaire was used. Quality of life scores for disease targeted areas and general health scores UWHI and DARU had good quality of social interactions and social functioning domain scores compared to the reference population. However, both cohorts had significantly worse burden of kidney disease scores than the reference population. Physical functioning domain scores in DARU cohort of patients was below that of the reference population. The domain of emotional role was significantly reduced in the DARU cohort of patients. Patient satisfaction scores were significantly low in both cohorts. In the Jamaican cohort, younger age, race, higher urea reduction ratio and higher serum haemoglobin predicted higher quality of life scores. Higher income bracket reported better quality of social interactions and energy scores. Higher dialysis staff encouragement and patient satisfaction scores were noted in those with health insurance coverage.	Quantitative score 0.78
Spence et al [33] 2010 Jamaica	A needs assessment survey of cancer patients in Jamaica. Mixed Methods Study	*n* = 159 (Patients = 81, caregivers = 51, health care professional = 20, informants of the local community = 7). They were interviewed using semi structured interviews. There were also 4 focus groups each consisting of 8–12 participants	**Patients** **Barriers to seeking treatment:** *1. Consultation experiences with health professionals* Themes of mistrust and poor communication with healthcare practitioners were found as barriers to seeking medical attention. Patients felt the long waiting times, lack of understanding of their diagnosis and disrespect from health professionals’ discouraged them from seeking medical attention. It was felt this may help explain why some people seek treatment from bush doctors and spiritual healers. These alternative practitioners provide quick and simple advice and were more approachable according to participants. *2. Folk wisdom/myths/lack of understanding person’s fears, lack of knowledge and belief in folk wisdom play a role in late presentation or delayed access to healthcare facilities for suspected cancer related illnesses*. A diagnosis of cancer is often viewed as an immediate “death sentence”. Beliefs such as “only evil people get cancer” and that cancer is a form of “god’s punishment” or cancer is a curse are held. These beliefs cause patients to believe that “obeah” (refers to “witchcraft, evil magic or sorcery by which supernatural power is invoked to achieve personal protection or the destruction of enemies”) was the way to treat these disorders. Some patients believe there is a stigma attached to certain cancers, for instance cervical cancer may be linked to sexual promiscuity or prostate cancer heralds threats to masculinity and sexuality. *3. Financial barriers* 70 % of the patients identified lack of financial resources as the single most important factor for non compliance with treatment regimes. **Needs of patients and caregivers** *1. Need for financial support*, 69 % of patients interviewed reported problems paying for cancer treatments.67 % state the inability at times to afford medications. A small percentage of patients identified that transportation costs hindered visits to health care facilities. Focus group participants suggested financial difficulties arise because the illness affects the ability of the patient or caregiver to work. Patients are also concerned about the long term financial stability of their family, so reluctant to put all resources towards their own healthcare. The inability to access affordable and nourishing foods were barriers to the patient’s ability to enjoy a fair quality of life. *2. Need for access to pain medication and other medicines*, 42 % of those experiencing pain or discomforts were not currently taking any form of prescription medication. 75 % of caregivers identified pain management as the most critical need of the person for whom they were caring. *3. Need for home care support* 31 % of the patients interviewed expressed a preference for home care, 46 % for in-hospital care and 21 % indicated no preference. They cited a preference for hospital care since the majority identified better care in the hospital and inadequate home care provisions. 1 % of caregivers stated a preference for their relative to be cared for at home. 4. *Need for emotional support and counselling and education*, Participants of the focus groups and alternative health practitioners spoke in detail about the need to offer ongoing emotional, spiritual and social support to patients and their caregivers. However, the patients (79 %) interviewed by the survey, suggested that they felt supported by family and community. Focus group members expressed the need for education to dispel misconceptions about cancer, to understand the diagnosis and different treatment options. **Needs of healthcare providers** Majority of the respondents thought that the capacity to deliver effective care to the terminally ill was impeded by breaches in government support at both regional and national levels. They felt that there were no strategies, protocols or policies in place for palliative or home based care in the region. They also felt there is a lack of appropriate training in palliative care. Many practitioners were poorly informed as to what palliative care is or what it could offer. They also pointed out restrictive government policies over access to certain analgesics as a challenge to deliver adequate pain relief.	Quantitative score:0.5 Qualitative score:0.8
Cleary et al [34] 2013 Anguilla Barbados Dominica Dominican Republic Jamaica St Lucia Trinidad and Tobago	To determine formulary availability and regulatory barriers to accessibility of opioids for cancer pain in the Caribbean. Cross Sectional Survey	The data were collected from physicians working in the field of palliative care. It was difficult to determine the number of participants who were from the Caribbean since the data from the Caribbean and Latin American were summated together. Data submitted from two or more field reporters from each country and rechecked by the principal investigator.	Formulary Availability of different opioids (formulations of codeine, immediate release morphine, controlled release oral morphine, injectable morphine, oral immediate release oxycodone, transdermal fentanyl and oral methadone) were looked at. The deficiencies in availability of the opioids and regulatory barriers were described below for each country presented in this paper. Anguilla does not have injectable morphine Dominica and Trinidad and Tobago reported to have no Immediate Release oral morphine Dominica has no codeine and controlled release oral morphine available Oral release oxycodone is not available in Barbados, Dominica, Jamaica and Trinidad and Tobago. Oral Methadone isn’t available in Dominica, St Lucia and Jamaica Transdermal fentanyl isn’t available in Anguilla, St Lucia, Dominica and Trinidad and Tobago Anguilla reports with the availability of the Immediate Release oral Morphine on the formulary, it was only available occasionally. Anguilla does not allow outpatient prescribing of opioids. In Anguilla, family doctors require special authority to prescribe opioids and surgeons are only allowed to prescribe in an emergency. Anguilla, Jamaica and St Lucia allow nurse prescribing with special permit. Pharmacists can prescribe opioids with special permit in Anguilla alone. In Jamaica and Dominican Republic, the limit of the prescription filled for opioids is 10-15 days. Jamaica, Barbados and Trinidad and Tobago have the use of stigmatizing terminology in the regulations for opioid analgesics. Jamaica, St Lucia and Trinidad and Tobago allowed Pharmacists to accept emergency prescriptions as well as for the pharmacist to correct technical errors. Anguilla, Dominican Republic, Jamaica and St. Lucia only allow dispensing of opioids from hospital pharmacies.	Quantitative score: 0.83
MacPherson et al [35] 2014 Antigua and Barbuda Barbados Grenada Jamaica Montserrat St Kitts and Nevis St Lucia St Vincent and the Grenadines Trinidad and Tobago	To document the availability of Hospice and palliation in the English speaking Caribbean. Cross Sectional Survey	*n* = 10 (Chief Medical Officers or their designees or presidents of their national medical association were asked to complete the survey from each country). The professions of these executives were not revealed.	**Description of hospices or palliative facilities and palliative care specialists amongst certain Caribbean Islands** Trinidad and Tobago has 2 private facilities (hospice or palliative facilities) and 2 palliative care specialists. St Vincent and the Grenadines has 1 public facility and 3 palliative care specialists. Jamaica has 1 public and 1 private facility and 2 specialists. Montserrat has 1 public facility and 1 specialist. Antigua and Barbuda and Barbados report 1 public facility and no specialist St Kitts and Nevis report 1 private facility and no specialist. Grenada and St Lucia report no facilities or specialist. The author suggested that 4 respondents have some form of home care provision to terminally ill patients. The names of these islands were not provided. *Pain Protocols* Antigua, Barbados, St Lucia and Trinidad and Tobago report having “a protocol for pain management, hospice or end of life care”. *Oral Morphine* All respondents indicated that oral morphine was medically “available to treat mild or severe pain” *Education on Palliation or Hospice* Author suggested five countries reported one or more “workshops or educational programs on palliative care.” These countries were not identified.	Quantitative score:0.57

## Results

**Fig. 1:**
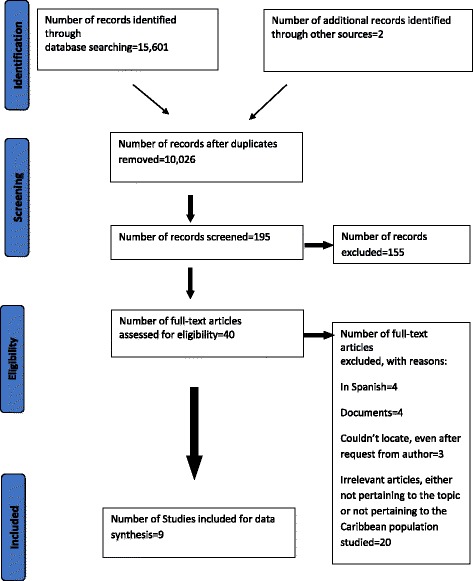
PRISMA flowchart

The search yielded a total of 10,026 references after duplicate references were removed. After application of the inclusion and exclusion criteria, the data of nine studies [27–35] were retained and synthesized in this review. The PRISMA flow chart representing this selection process is shown in Fig. 1. The characteristics of these nine studies are represented in Table 4.

### Countries reported

Four studies reported data from Jamaica [32–35], three each from Barbados [27, 34, 35] and Cuba [29–31], two studies each from Trinidad and Tobago [34, 35], St Lucia [34, 35] and Grenada [28, 35] and one study reported from each of the following islands: Antigua and Barbuda, Montserrat, St Kitts and Nevis, St Vincent and the Grenadines [35], Anguilla, Dominica and the Dominican Republic [34] . Please refer to Table 4 to view the studies in detail. There were no studies from Aruba, Bonaire, British Virgin Islands, Curaçao, Guadeloupe, Martinique, Saba, Saint Barthelemy, Saint Martin, Saint Eustatius, US Virgin Islands, The Bahamas, Turks and Caicos, Haiti and Cayman Islands. Regrettably, a few studies from Puerto Rico were written in Spanish and therefore did not meet our inclusion criteria.

### Study methods

Seven studies [27, 29–32, 34, 35] used quantitative methodological approaches, and cross sectional survey designs were utilized within these studies. One study used a mixed methods design with qualitative and quantitative components [33]. One study used a qualitative approach using interviews and focus groups [28].

### Topics reported

Eight studies, [27–34] reported palliative care needs in the Caribbean. Two studies [34, 35] briefly illustrated palliative care services in the Caribbean and only described them for certain islands. There were no intervention studies. All studies pointed out that palliative care is needed in the Caribbean. Outcomes of quality of life [31, 32], quality of care provided to patients with advanced cancer [29, 30] and patients’ preferences for place of care and place of death [28, 33] were described in six studies [28–33].

### Study sample sizes

Sample sizes from seven studies, [27–33] were able to be determined. There were 312 patients across four studies [27, 31–33], 75 caregivers from two studies [28, 33] and 104 health practitioners interviewed in four studies [28–30, 33]. One study from Jamaica [33] interviewed seven local community members and held four focus groups ranging from 8–10 participants for each group. A further study [34] interviewed physicians from Caribbean countries, however the actual numbers of physicians were difficult to identify since the results from the Caribbean countries were combined with physicians reports from Latin America. One study [35] identified ten participants from different Caribbean islands; however it was difficult to determine the professions of those interviewed.

### Diagnostic groups and health professionals

Diagnoses of the patients included in the review were patients with advanced cancer in two studies [31, 33], end stage renal disease [32] in one study, and moribund intensive care [27] patients from one another. Two studies included eight physicians and fourteen nurses [28, 33]. The specialties of these health professionals were not mentioned in the study. The health practitioners from two studies [29, 30] comprised the same study subjects (deciphered from methodology, data presented and authors of the papers) and presented percentages suggesting that respondents specialties were medical (44 %), surgical oncology (25 %), anesthesiology (13 %), radiotherapy (8 %), paediatric oncology (5 %), nursing (4 %) and other (1 %). Meta-analyses of quantitative data were not performed in this review since the studies were heterogeneous in nature.

### Thematic analysis

Eight themes arose from this review based on its broad scope . A significant number of themes were generated since the papers provided rich viewpoints from a diverse population. The themes generated in this review were, Theme 1- Patients need for appropriate pain control and access to analgesics, Theme 2- Patients need for support, Theme 3- Lack of knowledge of palliative care by health care professionals, Theme 4- Lack of trained staff and need for more staff in palliative care, Theme 5- Need for specialized palliative care services, Theme 6 - Deficiency in health care policy, Theme 7-Cultural beliefs of patients and Theme 8-Patients preferred place of care and death.

#### Theme 1 patients need for appropriate pain control and access to analgesics

Two studies [28, 33] reported patients and/or their caregivers view that there was a need for analgesics and also access to these medications. In one study [28], caregivers mentioned “she thought she was going to die because she had so much pain” and “he was really scared about that pain” when asked if the deceased suffered much pain. In another paper [33], seventy five percent of the caregivers identified pain management as the most critical need of the person for whom they were caring. Three papers [28, 33, 34] highlighted the issue of difficulty in accessing opioids in the Caribbean for cancer pain. One paper [34] drew attention to the opioid formulary deficiencies in some Caribbean islands that reduce access to some of these drugs. The paper illustrated the lack of particular opioids in some Caribbean islands, and restrictions enforced for prescribing and dispensing opioids. In addition, a study based in Cuba [29], health practitioners suggested that international restriction on the import and export of medications and the fear of diversion of opioids to the illegal market as two of the top barriers to optimal cancer care in that country.

#### Theme 2 patients need for support

The need for emotional, spiritual, financial and social support to patients and their caregivers were described in three papers [28, 32, 33]. Caregivers in Grenada [28], divulged the financial hardships secondary to medical and/or funeral costs for the deceased. They were unaware of any bereavement services such as counselling, financial assistance and home care assistance. One participant suggesting: “our system is not geared toward helping people; there is no social thing here to help you.” In Cuba [29] health practitioners noted that the affordability-of-care predicted the quality of advanced-cancer care.

Spirituality was mentioned by the majority of the participants in the survey in Grenada [28] as being important to them. A participant relates “I think the best solution with no money or anything is the lord in prayer.” They also identified that emotional support came from friends and family.

One study [31] used the Palliative Outcome Scale to discover that patients with advanced cancer in Cuba report their most burdensome problems as patient and family anxiety.

In Jamaica [32], results from a study on quality of life in end stage renal disease, attention was drawn to low patient satisfaction, general health, physical functioning, physical role and emotional role scores when compared to a reference population.

#### Theme 3 lack of knowledge of palliative care by health care professionals

Some of the physicians that participated in the needs assessment survey in Jamaica [33] poorly understood the meaning of palliative care and its constituents. In Cuba [29], health professionals identified that palliative care is not a priority in health care education as one of the top barriers to optimal cancer care.

#### Theme 4 lack of trained staff and need for more staff in palliative care

A cross sectional study [35] carried out amongst eight of the Caribbean islands included, found that there were five specialists in palliative care in total amongst three of these islands. In Jamaica, health care practitioners [33] believed there was not enough training in palliative care. Health care providers in Cuba [30] cited that the specialties of the provider were predictors of the quality of advanced cancer care. They also noted medical staff reluctance to prescribe opiates as a barrier to the quality of advanced cancer care. A study in an Intensive Care Unit in Barbados [27] reported that anesthesiologists and surgeons found it difficult to withdraw treatment even though there was an agreement of futility of treatment.

The survey participants in Grenada [28] voiced that there was not enough kindness from the nurses towards the deceased. The nurses in this same study allude to there being isolated cases of mistreatment of patients. Limited support services and the need for additional staff, for instance, the chaplain, counsellors and social workers were emphasised by the nurses interviewed in this study. Physicians who were interviewed conveyed concerns about the nurses’ low salaries, workload, long working hours and also difficulty in accessing patients’ homes to care for dying patients at home.

The participants in the Jamaican study [33] felt that there were communication issues between patients and health practitioners. They suggested that patients often do not understand what is being said to them by doctors. They felt disrespected and had lack of trust for the physicians.

#### Theme 5 need for specialized palliative care services

Of the eight Caribbean islands surveyed [35], two of the islands do not offer any palliative care facilities. Health care providers in Cuba [30] propose availability of end-of life services as the main predictor of quality of care.

#### Theme 6 deficiency in health care policy

In two of the papers [29, 33], health practitioners reported that palliative care appears not to be a priority in the formulation of health care policy. The participants in the Jamaican study went on to comment that “the capacity to deliver effective care to the terminally ill was curtailed by gaps in government support at both regional and national levels”[33]. This reinforced the absence of protocols and policies in the formation of palliative care services.

The paper which identified the greatest issues with health care policy is a report from the Global Opioid Policy Initiative (GOPI) [34]. This study highlights not only the absence of certain opioids in the formulary in some Caribbean islands, it also lists regulatory barriers for accessing opioids in these countries. A few barriers in some Caribbean islands were that physicians require special licence or permission to prescribe opioids, the limits in the number of days opioids can be dispensed, limitations on which pharmacies can dispense opioids and negative connotations of opioids in some countries, describing them as “drugs of addiction” or dangerous drugs”. Lack of health care resources such as pain relief and personnel were noted by physicians from Grenada [28].

Finally, legal considerations must also be considered since ICU practitioners in Barbados [27] voiced the fear of litigation if they were to withdraw futile therapy from a patient.

#### Theme 7 cultural beliefs of patients

Some participants in the study in Jamaica [33] considered that the belief in folk wisdom and the patients’ lack of knowledge had a part to play in their late presentation or unwillingness to present to health care facilities for suspected cancer. Some patients believe a diagnosis of cancer equals an “instant death sentence” and “only evil people get cancer”. By the same token, reports from the physicians in the Grenadian study [28] state Caribbean people “believe that illness is a God given destination, so they don’t mind suffering. They believe that maybe it was something they did, and it is a question of almost purification before they go beyond. So they accept it.” Since cancer was deemed to have spiritual roots, patients felt that witch doctors or “obeah” (witchcraft or evil magic) persons or “madda” were better able to diagnose and treat spiritual problems rather than a medical doctor [33]. Physicians in the Grenadian study [28] suggested with regards to herbal medication, the terminal and chronically ill put faith into that approach so that they would have explored all avenues of treatment.

Another point generated by the Jamaican study [33] was the stigmatization of certain cancers. For instance, a diagnosis of prostate cancer was seen as a threat to masculinity. The participants of the focus groups mentioned that education for patients with regards to clarifying misconceptions about cancer and addressing the myths was required to assist with stigmatization.

#### Theme 8 patients preferred place of care and death

The majority of caregivers in the Jamaican survey [33] suggested that they preferred to care for their relative at home. The patients in this survey cited the preference for hospital care followed by home care. They expressed the preference for hospital care since there were inadequate home care services. In Grenada [28], many of the deceased preferred to stay/die at home for different reasons as reported by their caregivers.

### Exploring relationships in the data

**Fig. 2:**
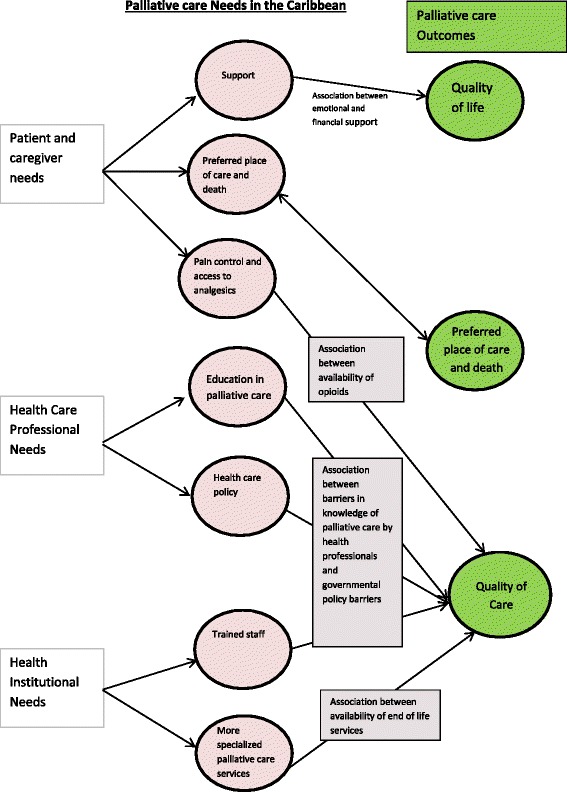
Demonstrating concept mapping of the themes extracted from the multiple studies included in this systematic review and their relationship with outcomes in palliative care generated from the studies

Idea webbing and concept mapping were used for exploring relationships in the data. This was chosen because of heterogeneity of the identified papers. The quantitative and qualitative data sets can be represented conceptually and link various findings from the individual papers [22]. Please see Fig. 2 for the model demonstrating the relationships from the studies included in this review.

**Fig. 3:**
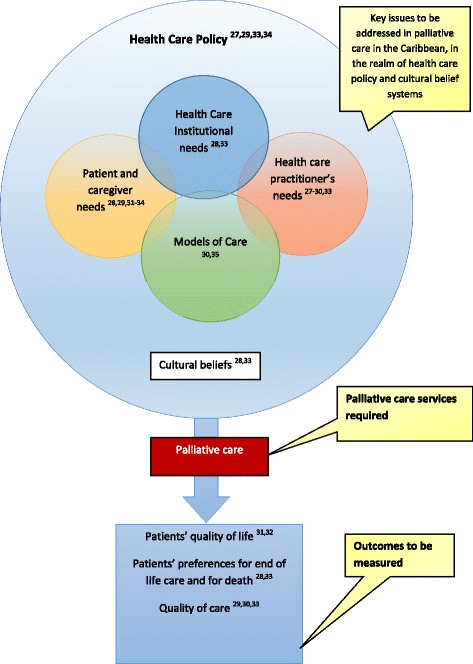
Idea webbing representing the possible relationships across studies and their integration of these ideas into a proposed conceptual model using abstract and inductive reasoning

Using the themes generated from the included studies, a model was also inductively formed from them and represented in Fig. 3. The use of idea webbing generated this second model and took a step further from Fig. 2 by attempting to make a connection between the themes induced from the different papers. The themes could be arranged into broad categories of needs, models of care, health care policy, outcome measures and cultural beliefs. The combination of health care needs and services are necessary for provision of palliative care. However, overarching health care policies and cultural beliefs must also be taken into consideration for an intervention such as palliative care.

## Discussion

This is the first systematic review of the peer reviewed literature on palliative care from the Caribbean. A literature review [36] of models of palliative care delivery globally was undertaken as part of the Barbados Palliative care Needs assessment project. This article, together with the survey of cancer patients, caregivers and health professionals carried out in Jamaica [33] included in this review, represent two types of needs assessment methods; comparative and corporate approaches.

The results from our review described patients’ needs and caregivers’ requests for pain relief, access to analgesics, holistic support and assistance with their wishes for preferred place of care at the end of life. Community members interviewed, echoed the need for emotional support and counselling for patients and their caregivers. They added that education on diagnosis and treatment options was necessary. Health care practitioners identified their needs for more education and training in palliative care. Moreover, a lack of healthcare policy regarding laws, formulary deficiencies and legal considerations were significant findings in this review. These findings highlight many issues in the provision of palliative care in the Caribbean. The needs underscore the importance of developing sustainable models of care in palliative care in the Caribbean since the demand for it is apparent.

The World Health Organization (WHO) Public Health Strategy describes four components or pillars which must be addressed for integration of palliative care into society [37]. Researchers with an interest in palliative care in Africa propose in addition to the four pillars of policy, education, drug availability and implementation that a fifth pillar of research activity is also required to support this strategy in Africa [38]. The recognition by regional and world health bodies of the importance of palliative care integration into the health systems of the developing countries is mammoth. Therefore, for palliative care to be integrated into the Caribbean health care system a lot of work still needs to be done at the level of the core pillars. It is known that the understanding of a population’s need for palliative care is crucial in planning services [39].

In order to plan palliative care services, health policy must be in place. Authors postulate that to contribute to effective policy, there must be a bridge in the gap in between theory, policy and practice [40]. The WHO Public Health Strategy provides the theory for integration of palliative care in health systems. The practice of palliative care in the Caribbean was only superficially described in the published literature. However, there is a notion of more established services in some of the Caribbean islands that is not documented in peer reviewed literature. In the process of reviewing literature for this research piece, apart from finding peer reviewed publications, documents such as opinion pieces, articles, and pamphlets were also discovered. Opinion pieces on services available in Trinidad and Tobago [41], surgical palliative care in Haiti [42], bereavement practices and their outcomes amongst religious and ethnic groups in the English speaking Caribbean [43] did not meet the inclusion criteria for this systematic review.

Trinidad and Tobago has a Palliative care Unit opened in 2014, which has not been reported in peer review literature as yet. Antigua has a hospice which has not been documented by the International Observatory on End of life care at Lancaster University in the mapping of global provision of palliative care. These are just a couple of examples which are known to the researcher that provide palliative care services and not acknowledged in research articles.

Although, outcomes (quality of life and quality of death) were alluded by the papers in this systematic review, it was reassuring to find a validated tool (the Palliative Outcome Scale :POS) was used in one of the studies included.

Health care practitioners from this review voiced the need for health care policy on palliative care. Although, all the themes found in this systematic review are extremely important, addressing policy appears to be a consistent principle amongst most themes. The seminal paper produced by the Global Opioid Policy Initiative on formulary availability and regulatory barriers to accessibility of opioids in the Caribbean and Latin America tackles many crucial issues that are correlated with palliative care needs in the Caribbean [34]. One author from Grenada suggests the need for stakeholder consultation to produce ‘locally effective and sustainable’ services in palliative care [44]. She recognizes that there is a need for palliative care in the Caribbean; however its provision must be in a feasible framework of cost, cultural beliefs and the national health systems available in the Caribbean [44].

The idea of successful integration of palliative care into the health care system of the Caribbean stimulated the production of the model portrayed in Fig. 3. However, as more research is conducted in the Caribbean this model can be developed further.

### Strengths and limitations

The studies included for this systematic review were retained following appraisal against the inclusion and exclusion criteria by one reviewer, a potential bias. In mitigation, a second senior researcher was consulted if any doubts were encountered in retaining or rejecting a study for the review.

Overall, the studies were quite similar in design, with the majority utilizing cross sectional surveys. Cross sectional studies are well suited to the research question since they aim to be descriptive, determine prevalence and reveal associations with regards to outcomes. There was one qualitative and one mixed methodology cross sectional survey design. Sample sizes across individual studies were noted to be small. The numbers could not be pooled due to study/sample heterogeneity.

The QualSyst tool was an appropriate tool for assessing study quality; however it did lack the ability to identify specific biases in the papers. This may have resulted in papers having inflated quality grades. On reflection, other tools could have been considered especially for assessment of the qualitative studies. For instance, the Qualsyst tools were designed for the purpose of producing a threshold for inclusion of a study into a systematic review based on quality. However, a question posed by this review was to appraise the quality of available evidence. A decision was made for assessing the scores from the qualitative papers by dividing them into two main groups based on the cut-off point provided by the authors of the tool: adequate quality and low quality. There was limited application of the tool in the evidence base for assessing qualitative work which therefore restricted its validity and reliability for use in this type of research piece. The Qualsyst tool when used for assessing the methodological quality of quantitative studies was found to be applied in a few studies in the literature base. This allowed for an established way of using the tool by grading the literature.

In general, biases and methodological flaws were observed in many of the studies, which included: selection bias with the use of non-probability sampling, reporting bias for those studies that collected narratives from participants, and observer bias where data collectors may have reported favourably or unfavourably on certain outcomes subliminally or interpreted the narrative incorrectly. In addition, the characteristics of the participants who did not respond to the survey were sometimes not presented, introducing potential non-participant bias. Measurement bias occurred in a few studies since non validated tools were used.

In addition, some papers were excluded since they were in Spanish and others could not be retrieved, instituting publication bias and also the inability to include possibly important information.

Non probability sampling methods and where the sample was drawn from may have created unrepresentative samples for ethnicity, education level, socio economic status and abode (whether urban or rural areas) in most of the studies included in this review. In addition, physicians who would have given information on their countries palliative care services, policies and opioid records may not have reported fully accurate or recent data. The studies included in this review ranged in publication from 2003-2014. The paper published in 2014 [35] describes palliative care services in the Caribbean; however, many of the earlier published papers helped shape the palliative care needs in the Caribbean. These needs may have changed over time.

Furthermore, five out of the nine papers included in this review focused on a diagnosis of cancer, with one on end stage renal disease. Therefore, the needs of patients with other disease processes requiring palliative care at some point during their illness trajectory were not described.

## Conclusions

This systematic review has revealed that the peer reviewed literature offers little evidence on palliative care needs of the Caribbean population. The available evidence was broadly divided into health care practitioner’s needs, patients’ needs and health care institutional needs. They included patients’ needs for access to analgesia, preferred place of care /death and multi-dimensional aspects of support needed for patients and their caregivers. Health care practitioners spoke about their need for health policy and education in palliative care. In addition, needs of more trained staff and the services in palliative care were cited as institutional needs. The models of care existent in the Caribbean were not well described in the peer reviewed literature. There were no intervention studies. It is timely to now develop and evaluate evidence-based palliative care services in the Caribbean.

## Abbreviations

CARICOM: The Caribbean Community
CINAHL: Cumulative Index to Nursing and Allied Health Literature
COPD: Chronic Obstructive Pulmonary Disease
CRD: Centre for Reviews and Dissemination
EMBASE: Excerpta Medica Database
GMC: General Medical Council
HIV/AIDS: Human Immunodeficiency Virus and Acquired Immune Deficiency Syndrome
LMIC: low and middle income countries
MEDLINE: Medical Literature Analysis and Retrieval System
MeSH: Medical Subject Heading
NCDs: Non Communicable Diseases
NHS: National Health Service
PAHO: Pan American Health Organization
POS: Palliative Outcome Scale
PRISMA: Preferred Reporting Items for Systematic Reviews and Meta-Analyses
St: Saint
UNAIDS: The Joint United Nations Programme on HIV and AIDS
US: United States
WHO: World Health Organization
